# Inferring SARS-CoV-2 functional genomics from viral transcriptome with identification of potential antiviral drugs and therapeutic targets

**DOI:** 10.1186/s13578-021-00684-4

**Published:** 2021-09-08

**Authors:** Xu Pan, Xin Li, Shangwei Ning, Hui Zhi

**Affiliations:** 1 College of Bioinformatics Science and Technology, Harbin Medical University, Harbin, 150081 China; 2 Department of Dermatology, Xiangya Hospital, Central South University, Changsha, 410008 China

## Abstract

Coronavirus disease 2019 (COVID-19) is an emerging infectious disease caused by the severe acute respiratory syndrome coronavirus 2 (SARS-CoV-2), and has posed a serious threat to global health. Here, we systematically characterized the transcription levels of the SARS-CoV-2 genes and identified the responsive human genes associated with virus infection. We inferred the possible biological functions of each viral gene and depicted the functional landscape based on guilt-by-association and functional enrichment analyses. Subsequently, the transcription factor regulatory network, protein–protein interaction network, and non-coding RNA regulatory network were constructed to discover more potential antiviral targets. In addition, several potential drugs for COVID-19 treatment and prevention were recognized, including known cell proliferation-related, immune-related, and antiviral drugs, in which proteasome inhibitors (bortezomib, carfilzomib, and ixazomib citrate) may play an important role in the treatment of COVID-19. These results provided novel insights into the understanding of SARS-CoV-2 functional genomics and host-targeting antiviral strategies for SARS-CoV-2 infection.

**Supplementary Information:**

The online version contains supplementary material available at 10.1186/s13578-021-00684-4.

Dear Editor,

The rapid spread of coronavirus disease 2019 (COVID-19) caused by the severe acute respiratory syndrome coronavirus 2 (SARS-CoV-2) has resulted in worldwide concern. High-throughput sequencing studies have identified SARS-CoV-2 as a positive-sense RNA virus with a genome size of 29,903 nucleotides. Similar to other coronaviruses, a set of subgenomic mRNAs are produced during the SARS-CoV-2 replication, including the structural protein mRNAs (*S*, *M*, *E*, and *N*) and other small accessory protein mRNAs (*ORF1ab*, *ORF3a*, *ORF6*, *ORF7a*, *ORF7b*, *ORF8*, and *ORF10*). These subgenomic mRNAs have common eukaryotic mRNA-like structures, such as the 5′ leader sequence and 3′ poly-A tail structure [1]. However, to date, the function of viral genes in COVID-19 infection remains unclear.

To systematically investigate the viral transcriptome, we first obtained RNA-seq datasets of SARS-CoV-2-infected cells from GSE147507. The dataset included three independent biological replicates of primary human lung epithelium NHBE cells, human lung adenocarcinoma Calu3 and A549 cells, and A549 cells transduced with a vector expressing human ACE2. All cells were mock-treated or infected with SARS-CoV-2 (USA-WA1/2020) at an MOI (multiplicity of infection) of 2 for 24 h, except for the transduced A549 cells, which were treated at an MOI of 0.2. We next quantified human and viral gene expression abundance, respectively. We aligned the raw reads to the GENCODE GRCh38 human genome assembly using Bowtie2 and normalized the counts of human genes to TPM (Transcripts Per Million) using RSEM. Next, the unaligned reads were assigned to the SARS-CoV-2 reference genome (NCBI Reference Sequence: NC_045512.2). Viral genes were quantified using the viGEN pipeline (Additional file 1). Compared with other viral genes, the *nucleocapsid* (*N*) gene expression level was the highest and *ORF7b* was the lowest in all SARS-CoV-2-infected cells (Fig. 1A). Moreover, we identified differentially expressed human genes in five SARS-CoV-2-infected and mock-infected control experiments using the paired Student’s *t* test. Genes with *P *value  <  0.05 in more than three experiments were recognized as differentially expressed genes (n  =  185) (Fig. 1B; Additional file 2: Table S1).

**Fig. 1:**
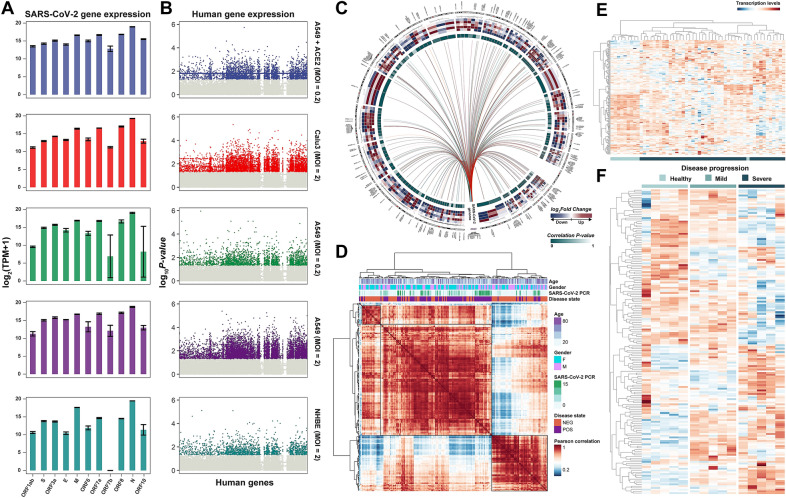
Overview of SARS-CoV-2 genes and human SR genes in SARS-CoV-2 infected human cells. **A** Viral gene expression levels in SARS-CoV-2-infected human cells. Error bars show mean  ±  SE of results of replicates. **B** Differentially expressed human genes responding to virus infection. The *x*-axis shows all the human genes. The *y*-axis shows the -log_10_(*P *value) based on the paired t-test between SARS-CoV-2 infected and mock-infected cells. **C** Circosplot showing the SR genes of each viral gene. Circos plot’s tracks, from outer to inner, are the heatmap of log_2_ Fold Change between SARS-CoV-2- and mock- infected cells in GSE147507, and the *P *values of Spearman’s correlation test between SR genes and RT-PCR results of SARS-CoV-2 in GSE156063. **D** Hierarchical clustering of the pairwise correlation of SR genes (rows and columns) in GSE156063. **E** Heatmap showing the clustering results based on SR genes among healthy and severe COVID-19 patients in GSE171110. **F** Heatmap showing the expression levels of SR genes among healthy, mild, and severe COVID-19 patients in GSE164805

To better understand the host transcriptional response to SARS-CoV-2, we interrogated the viral gene-related human genes based on the linear regression model, which was constructed between the transcription levels of differentially expressed human gene *i* (H) and viral gene *j* (V) and corrected for the effect of cell types and MOI as co-variables, i.e., ${H}_{i}\sim \alpha *{V}_{j}+\beta +\varepsilon$ (Additional file 1). Human genes with *P * <  0.05 were identified as the responsive genes (SR genes) associated with SARS-CoV-2 infection at the transcriptional level (n  =  180; Fig. 1C; Additional file 2: Table S1). To validate the association between SR genes and SARS-CoV-2, we tested the correlation between the SR gene transcription levels and SARS-CoV-2 concentration detected by RT-PCR in a COVID-19 cohort of 93 patients (GSE156063) (Fig. 1C). The results showed that the transcription levels of 123 SR genes in total varied with the concentration of SARS-CoV-2 (*P * <  0.05, Spearman’s correlation test). Furthermore, we calculated the coupled correlation coefficient among COVID-19 patients and healthy controls based on the transcription levels of SR genes and performed a hierarchical clustering analysis, which showed that COVID-19 patients tended to be clustered together (Fig. 1D, Additional file 1). The same result was also observed for distinguishing healthy individuals and patients with severe disease in a different COVID-19 cohort (GSE171110), suggesting that the dysregulation of SR genes might play an important role in COVID-19. Remarkably, in a dataset on COVID-19 disease progression (GSE164805), we found that transcription levels of SR genes also presented a dynamic variable trend with the progression of COVID-19 (from healthy to mild to severe) (Fig. 1E). These results highlighted the potential function of SR genes in response to SARS-CoV-2 infection.

Subsequently, we inferred viral functional genomics by performing functional enrichment analysis of the SR genes for each viral gene based on “guilt-by-association” (GBA) (Additional file 1). GBA states that genes which are associated are more likely to share similar functions [2]. Therefore, we could establish connections between each viral gene and host biological process based on the enriched functional terms of the viral SR gene (*P * <  0.05, hypergeometric test). Furthermore, we constructed the functional landscape of the host response to SARS-CoV-2 infection and similar functional terms were clustered (Fig. 2A). The SARS-CoV-2 genes extensively affected various cellular programs, such as immune response, metabolic processes, and signaling pathways (Table 1). Notably, one of the functional annotations for the viral spike (S) gene enriched in the reproductive system-related terms (GO:0048608 reproductive structure development, GO:0003006 developmental process involved in reproduction, and GO:0008585 female gonad development), indicated that S gene-related SR genes might be engaged in reproductive system-related biological processes. The S protein can help SARS-CoV-2 entry into host cells by interacting with cell surface entry factors, ACE2 and NRP1, to mediate the cell membrane fusion process (Fig. 2B) [3]. We found that both ACE2 and NRP1 were generally expressed at higher levels in the human reproductive system than in other organs at the transcriptome and proteome levels, implying a high risk of SARS-CoV-2 infection in the reproductive system (Fig. 2C). In addition, some tissue-specific expression of S gene-related SR genes in the reproductive system, e.g., *Testis Tissue Sperm-Binding Protein Li 44a* (*WDR77*), *Testis-Specific Gene A2 Protein* (*RSPH1*), *Follicle-Stimulating Hormone-Releasing Protein* (*INHBA*), suggested that the viral S protein might affect reproductive system-related processes (Fig. 2D). Recently, some case and autopsy reports have also demonstrated an involvement of the reproductive system in patients with COVID-19 [4, 5]. Although it is unclear whether the reproductive system of patients functioned normally prior to SARS-CoV-2 infection, the evidence prompts that we should be aware of any possible impact of SARS-CoV-2 on the reproductive system. As SARS-CoV-2 has evolved into a long-term problem, some S protein-based vaccines are widely used to prevent COVID-19. There are currently four main COVID-19 vaccines available: whole virus, protein subunit, viral vector, and nucleic acid (mRNA). Except for the whole virus vaccine, which is produced by inactive pathogens, the other three vaccines are designed and manufactured on the basis of the viral S protein. They all work by exposing the body to molecules from the target pathogens to trigger host immune response, however, they did not contain the live components (for whole virus, nucleic acid, and protein subunit vaccines) or produce the complete SARS-CoV-2 structure (for viral vector vaccine). Therefore, these vaccinations should be safe to develop immunity against SARS-CoV-2 infection and are unlikely to affect the reproductive system. Nevertheless, as these results are based on reasonable speculation and retrospective analysis, systematic validation in a large sample cohort is still required.

**Fig. 2:**
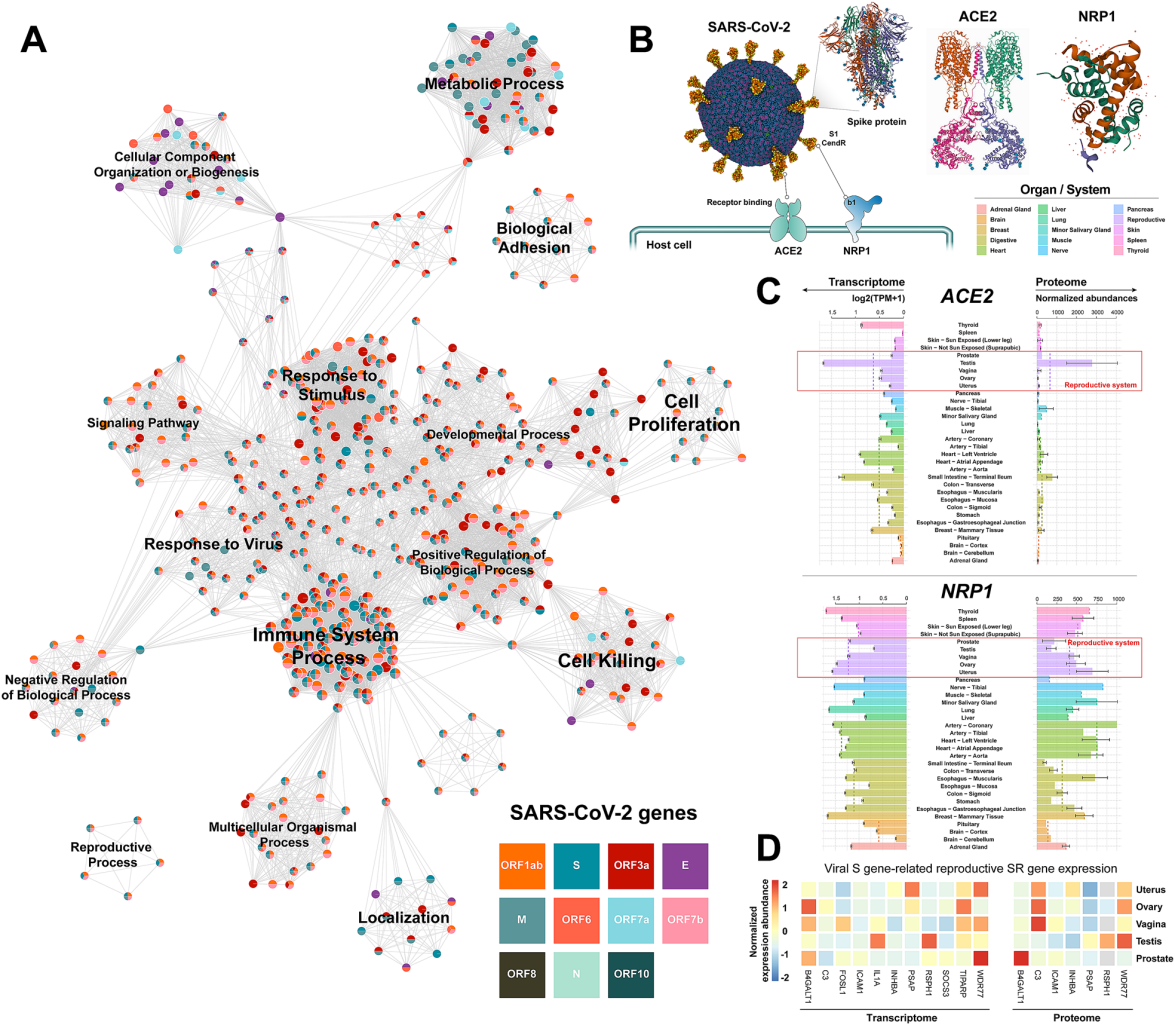
Inferring SARS-CoV-2 functional genomics. **A** The functional landscape of the SARS-CoV-2 genome based on Metascape. Each node represents a functional term from the KEGG Pathway, GO Biological Processes, and Reactome Gene Sets. **B** Illustration showing SARS-CoV-2 entry into the host cell via the S protein-mediated cell membrane fusion process. The SARS-CoV-2 structure is from “Mol*” (https://molstar.org/). The full 3D view of protein structure is available at PDB database (https://www.rcsb.org/). **C** Barplot showing mRNA and protein expression levels of ACE2 and NRP1 in human organs or systems. The mean expression level of each organ/system is marked with a dashed line. **D** Heatmap showing the mRNA and protein expression abundance of viral S gene-related reproductive SR genes in the human reproductive system

**Table 1:** The main biological processes of each SARS-CoV-2 gene

SARS-CoV-2 gene	Main biological processes in Gene Ontology
All genes	GO:0008152 metabolic process, GO:0032502 developmental process, GO:0071840 cellular component organization or biogenesis
ORF1ab	GO:0051179 localization
ORF7a	GO:0002376 immune system process
S, E	GO:0002376 immune system process, GO:0023052 signaling, GO:0022414 reproductive process, GO:0050896 response to stimulus
N	GO:0001906 cell killing
M	GO:0048511 rhythmic process, GO:0051704 multi-organism process

To better understand the response mechanisms within SARS-CoV-2 host cells, we performed an integrative network analysis (Additional file 1). First, we constructed the viral gene-SR gene- transcription factors (TFs) regulatory network, which consisted of five viral genes, 27 TFs, and 50 SR genes (Fig. 3A; Additional file 2: Table S2). These TFs might be involved in the regulation of SR gene expression in SARS-CoV-2 infected cells, where TFs NFKB1, RELA, and STAT3 have been demonstrated could respond to COVID-19 by previous studies [3]. Next, we established the protein–protein interaction (PPI) network, which included 236 interaction relationships between 117 SR genes (Fig. 3B). We found the *Complement component 3* (*C3*) gene was a hub node in the PPI network. *C3* was identified as the SR gene of viral *ORF3a*, *ORF7a*, *S*, *E*, and *N* genes, suggesting that its transcription levels might vary with these viral gene expression levels. In addition, the *C3* gene could interact with 14 other SR genes (Fig. 3B). Recent studies have suggested that complement C3 activation is an initial effector mechanism that contributes to thromboinflammation and organ damage in COVID-19. Indeed, patients with severe acute respiratory distress syndrome (ARDS) caused by COVID-19 pneumonia have also been safely and successfully treated with AMY-101, a compstatin-based complement C3 inhibitor [4]. These results highlighted the potential value of identifying SR genes in the development of antiviral treatment strategies. Besides, we constructed a non-coding RNA-SR gene co-expression network in COVID-19 patient peripheral blood (GSE166552), which consisted of 460 co-expression relationships between 125 ncRNAs (99 lncRNAs and 26 circRNAs) and 55 SR genes (Fig. 3C). Some hub circRNAs and lncRNAs were identified, which may be important in screening novel blood biomarkers of COVID-19 (Additional file 2: Table S3).

**Fig. 3:**
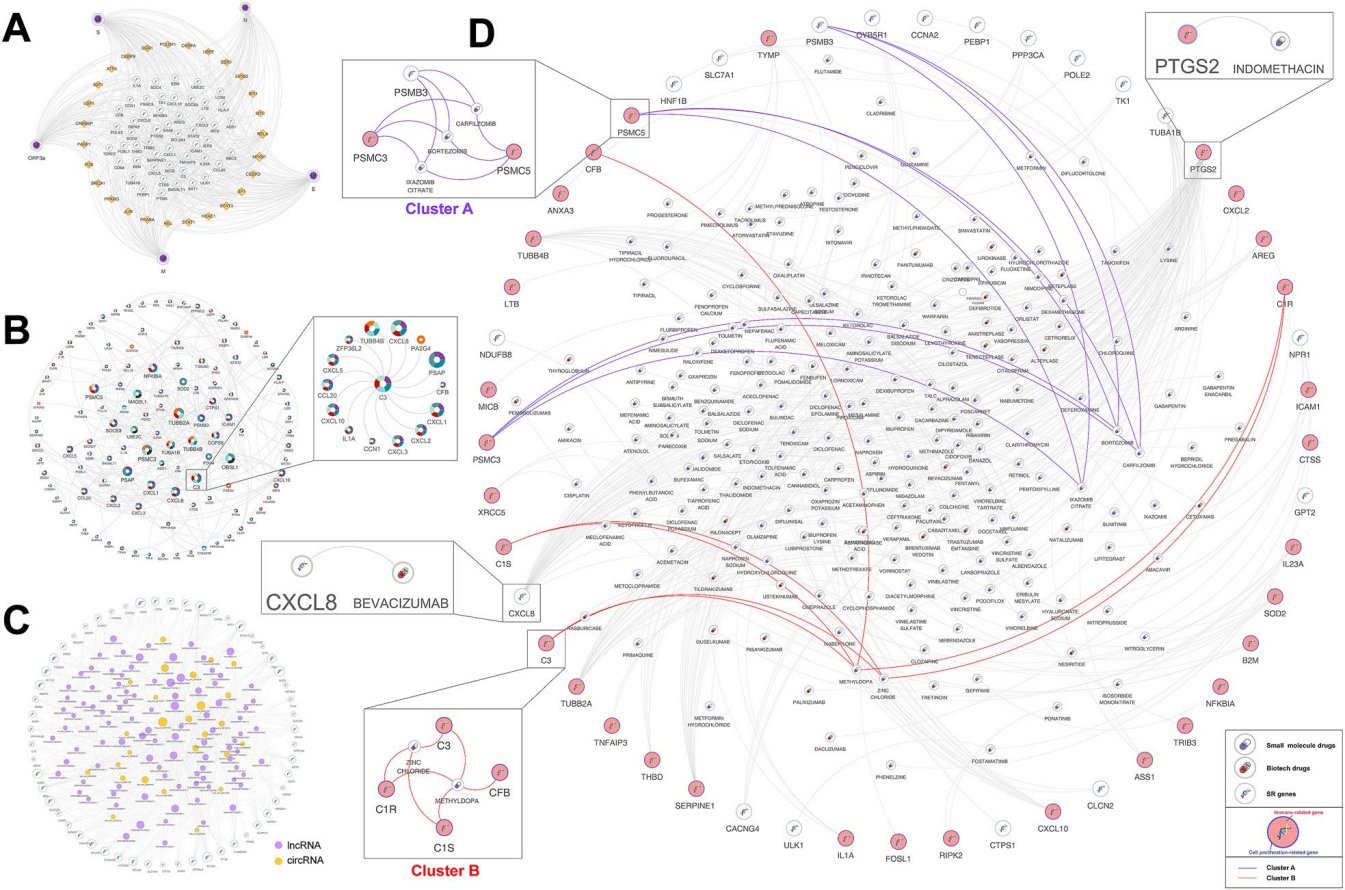
Integrative networks analysis of SARS-CoV-2 infected cells. **A** Viral gene-SR gene-TF regulatory network. **B** The PPI networks among SR genes. The node size is determined by the degree. Pie chart showing the association between viral genes and each SR gene. **C** The lncRNA/circRNA-SR gene co-expression network. The node size is determined by the degree. LncRNAs are shown in purple, while circRNAs are shown in orange. **D** Drug-SR gene interaction network. Small molecule drugs and biotech drugs are shown, respectively. For SR genes, the immune-related genes are shown in red, and cell proliferation-related genes are marked in blue. The drug-gene clusters identified by network clustering analysis are connected by different colors

In addition, we constructed the drug-SR gene interaction network to examine potential antiviral drugs (Fig. 3D; Additional file 2: Table S4). The networks contained 48 SR genes and 204 FDA-approved drugs, some of which have shown clinical efficacy for the treatment of COVID-19. For example, dexamethasone and methylprednisolone have been widely used to treat COVID-19 with promising benefits [5]. Indomethacin, a *PTGS2* inhibitor (SR gene *PTGS2* had been reported as a pro-viral host factor of SARS-CoV-2 [6]), has been used in several clinical trials for the treatment of COVID-19 and has shown promising clinical efficacy [7]. Bevacizumab, a monoclonal anti-vascular endothelial growth factor antibody, which exhibits immunomodulatory effects and has been used in combination with antineoplastic agents for the treatment of multiple cancers, has also been shown to have high clinical benefits for patients with severe COVID-19 in a single-arm trial (NCT04275414) [8]. We subsequently recognized some candidate drugs for SARS-CoV-2 infection based on the network cluster analysis, which resulted in two drug-gene clusters (Table 2; Additional file 1). Cluster A contained three proteasome inhibitors (bortezomib, carfilzomib, and ixazomib citrate), in which Carfilzomib was identified as a suitable candidate for the treatment of COVID-19 by computational drug repurposing studies [9]. Cluster B consisted of two potential drugs zinc chloride and methyldopa. Zinc, acting an immune booster, may prevent SARS-CoV-2 infection. Previous studies have indicated that zinc plays an important role in immune system development, and that increased intracellular zinc concentrations efficiently impair replication in a number of RNA viruses [10]. Several retrospective analyses of COVID-19 patients have also indicated that zinc deficiency is associated with a prolonged hospital stay and increased mortality [11, 12]. Thus, together, these findings suggested that zinc might be useful in preventing SARS-CoV-2 infection. In summary, SR gene-associated drugs provided novel insights into the development of antiviral treatment and prevention strategies in the post-COVID-19 era.

**Table 2:** List of potential drugs for SARS-CoV-2 infection based on the network cluster analysis

Clusters	SARS-CoV-2 gene	SR gene	Drug	Interaction types
Cluster A	S, E, M, N, ORF7a	C3	Zinc chloride	Inhibitor, ligand
		C3	Methyldopa	Unknown
	S, E, N, ORF3a	C1R	Zinc chloride	Modulator
		C1R	Methyldopa	Unknown
	S, E, N, ORF3a	C1S	Zinc chloride	Modulator
		C1S	Methyldopa	Unknown
	S, E, M, N, ORF3a	CFB	Methyldopa	Unknown
Cluster B	S, E, M	PSMB3	Bortezomib	Inhibitor
		PSMB3	Carfilzomib	Inhibitor
		PSMB3	Ixazomib citrate	Inhibitor
	S, E, N, ORF1ab, ORF3a	PSMC5	Carfilzomib	Inhibitor
		PSMC5	Bortezomib	Inhibitor
		PSMC5	Ixazomib citrate	Inhibitor
	M, ORF1ab, ORF7a, ORF8	PSMC3	Carfilzomib	Inhibitor
		PSMC3	Bortezomib	Inhibitor
		PSMC3	Ixazomib citrate	Inhibitor

## Supplementary Information

**Additional file 1: **Supplementary Methods.

**Additional file 2: Table S1. **The differentially expressed human genes and SR genes in SARS-CoV-2-infected human cells. **Table S2. **TFs that significantly associated with viral genes. **Table S3. **The co-expression relationships between ncRNAs and SR genes in COVID-19. **Table S4.** Potential host-targeted antiviral drugs associated with SR genes.

## Data Availability

All data analysed during this study are included in the article.

## Abbreviations

COVID-19: Coronavirus disease 2019
SARS-CoV-2: Severe acute respiratory syndrome coronavirus 2
SR gene: Human genes responding to SARS-CoV-2 infection
