# Supplementary material for: Inferring SARS-CoV-2 functional genomics from viral transcriptome with identification of potential antiviral drugs and therapeutic targets
Source: Cell Biosci. 2021 Sep 8;11:171. doi: 10.1186/s13578-021-00684-4 (PMC8424170; doi:10.1186/s13578-021-00684-4)
Supplement: Supplementary file 1 — Additional file 1: Supplementary Methods. [file 13578_2021_684_MOESM1_ESM.docx]

**Inferring SARS-CoV-2 functional genomics from viral transcriptome with identification of potential antiviral drugs and therapeutic targets**

Pan *et al.*

**Supplementary Methods**

**Data acquisition**

We downloaded the RNA-seq datasets of SARS-CoV-2 infected cells from GSE147507 [1]. The dataset included three independent biological replicates of primary human lung epithelium (NHBE), A549 and Calu3 cells that were mock treated or infected with SARS-CoV-2 (USA-WA1/2020) at an MOI (multiplicity of infection) of 2 for 24 h. The complete viral genome was downloaded from NCBI (NCBI Reference Sequence: NC_045512.2). The human genome assembly (GRCh38) was downloaded from GENCODE [2].

**Quantification of human gene and viral gene expression**

First, Trimmomatic [3] was used for quality control of the raw reads. Next, the human RNA reads were aligned to the human reference genome using Bowtie2 [4] and RSEM [5]. The unaligned reads were taken and aligned to the SARS-CoV-2 reference file (NCBI Reference Sequence: NC_045512.2) using Bowtie [4]. Finally, quantification of the viral genes was carried out using viGEN [6].

**Differential expression analysis**

We assessed the statistical significance of differentially expressed human genes between SARS-CoV-2-infected and mock-infected cells in five control experiments using the paired Student’s *t*-test. Genes with *P*-value < 0.05 in more than three experiments were considered to be differentially expressed.

**Identification of SR genes**

To identify the responsive human genes associated with SARS-CoV-2 infections, we constructed a linear regression model to link each viral gene to human genes, i.e.,

$H_{i}\sim\alpha*V_{j}+\beta+\varepsilon$,

where, H and V represent the SR gene i and viral gene j expression, respectively; $\beta$ denotes the co-variables (cell types and MOI). Human genes with *P*-value < 0.05 were identified as responsive genes (SR genes) associated with SARS-CoV-2 infection at the transcriptional level (Table S1).

**Clustering analysis**

We first calculated the coupled correlation coefficients among COVID-19 and healthy samples (GSE156063) based on the transcription levels of SR genes using Spearman’s correlation test. The coefficients were used to perform hierarchical clustering analysis.

**Functional enrichment and guilt-by-association analysis**

We performed functional enrichment analysis in Metascape for the SR genes of each viral gene [7]. The pathway and process enrichment analyses were carried out using the following ontology sources: KEGG Pathway, GO Biological Processes, and Reactome Gene Sets. Metascape used the hypergeometric test and *P*-value corrected by the Benjamin-Hochberg algorithm to identify significantly enriched ontology terms. Then, we employed the guilt-by-association method to establish the connections between each viral gene and host biological process, i.e., utilizing the enriched functions of the SR genes to infer the possible functions of the viral genes.

**Integrated network analysis of SR gene associations**

**Viral gene-SR gene-TF regulatory network**

We firstly enriched the SR genes of each viral gene into the downstream genes of human TFs using the hypergeometric test (Table S2). The TF downstream genes were obtained from TRRUST [8]. TFs whose downstream genes significantly overlapped with viral gene-related SR genes (*P*-value < 0.05) were used to construct the viral gene-SR gene-TF regulatory network. Network visualization and analysis were performed in Cytoscape [9].

**Protein-to-protein interaction network**

We integrated the direct physical interactions protein interactome (BioGRID [10]) between SR genes to construct the PPI network. The hub SR gene was then identified as a node of degree more than ten. Network visualization and analysis were performed in Cytoscape [9].

**The ncRNA-SR gene co-expression network**

We first identified the differentially expressed human lncRNAs (DE-lncRNAs) and circRNAs (DE-circRNAs) between COVID-19 and normal samples in GSE166552 ($\left| \log_{2} FC \right|>1$, *P*-value < 0.01, Student’s *t*-test). Subsequently, we recognized co-expressed lncRNAs and circRNAs with the SR genes in the DE-lncRNAs and DE-circRNAs using Spearman’s correlation test [11]. Then, the raw *P*-value ($P_{r}$) was corrected for multiple hypotheses using a permutation approach: keeping the SR gene expression constant, randomly producing the lncRNA/circRNA expression 1,000 times in normal distribution, and recalculating the Spearman’s correlation. The set of permutation *P*-values ($P_{p}$) were used to calculate the empirical *P*-value ($P_{e}$) as follow:

$$P_{e}=\frac{num\left( P_{p}\leq P_{r} \right)+1}{1001}$$

Finally, co-expressed lncRNA/circRNA-SR gene pairs with $Rho>0.7$ and $P_{e}<0.01$ were used to construct the ncRNA-SR gene co-expression network (Table S3). Network visualization and analysis were performed in Cytoscape [9].

**Drug-SR gene interaction network**

The drug-SR gene interaction network was constructed based on the drug-gene interactome data from DGIdb [12]. Next, we identified the key drug-gene interaction clusters by employing the “Molecular Complex Detection” (MCODE) algorithm. Network visualization and analysis were performed in Cytoscape [9].

**Reference**

1. Blanco-Melo D, Nilsson-Payant BE, Liu W-C, Møller R, Panis M, Sachs D, Albrecht RA, tenOever BR: **SARS-CoV-2 launches a unique transcriptional signature from in vitro, ex vivo, and in vivo systems.** *bioRxiv* 2020**:**2020.2003.2024.004655.

2. Harrow J, Frankish A, Gonzalez JM, Tapanari E, Diekhans M, Kokocinski F, Aken BL, Barrell D, Zadissa A, Searle S, et al: **GENCODE: the reference human genome annotation for The ENCODE Project.** *Genome Res* 2012, **22:**1760-1774.

3. Bolger AM, Lohse M, Usadel B: **Trimmomatic: a flexible trimmer for Illumina sequence data.** *Bioinformatics* 2014, **30:**2114-2120.

4. Langmead B, Salzberg SL: **Fast gapped-read alignment with Bowtie 2.** *Nat Methods* 2012, **9:**357-359.

5. Li B, Dewey CN: **RSEM: accurate transcript quantification from RNA-Seq data with or without a reference genome.** *BMC Bioinformatics* 2011, **12:**323.

6. Bhuvaneshwar K, Song L, Madhavan S, Gusev Y: **viGEN: An Open Source Pipeline for the Detection and Quantification of Viral RNA in Human Tumors.** *Front Microbiol* 2018, **9:**1172.

7. Zhou Y, Zhou B, Pache L, Chang M, Khodabakhshi AH, Tanaseichuk O, Benner C, Chanda SK: **Metascape provides a biologist-oriented resource for the analysis of systems-level datasets.** *Nat Commun* 2019, **10:**1523.

8. Han H, Cho JW, Lee S, Yun A, Kim H, Bae D, Yang S, Kim CY, Lee M, Kim E, et al: **TRRUST v2: an expanded reference database of human and mouse transcriptional regulatory interactions.** *Nucleic Acids Res* 2018, **46:**D380-d386.

9. Shannon P, Markiel A, Ozier O, Baliga NS, Wang JT, Ramage D, Amin N, Schwikowski B, Ideker T: **Cytoscape: a software environment for integrated models of biomolecular interaction networks.** *Genome Res* 2003, **13:**2498-2504.

10. Oughtred R, Stark C, Breitkreutz B-J, Rust J, Boucher L, Chang C, Kolas N, O’Donnell L, Leung G, McAdam R, et al: **The BioGRID interaction database: 2019 update.** *Nucleic Acids Research* 2019, **47:**D529-D541.

11. Wu Y, Zhao T, Deng R, Xia X, Li B, Wang X: **A study of differential circRNA and lncRNA expressions in COVID-19-infected peripheral blood.** *Scientific reports* 2021, **11:**7991-7991.

12. Cotto KC, Wagner AH, Feng YY, Kiwala S, Coffman AC, Spies G, Wollam A, Spies NC, Griffith OL, Griffith M: **DGIdb 3.0: a redesign and expansion of the drug-gene interaction database.** *Nucleic Acids Res* 2018, **46:**D1068-D1073.
